# Green Synthesis of CuO Nanoparticles—Structural, Morphological, and Dielectric Characterization

**DOI:** 10.3390/ma17235709

**Published:** 2024-11-22

**Authors:** Joana Neiva, Zohra Benzarti, Sandra Carvalho, Susana Devesa

**Affiliations:** 1 CEMMPRE, ARISE, Department of Mechanical Engineering, University of Coimbra, Rua Luís Reis Santos, 3030-788 Coimbra, Portugal; joanaluisneiva@gmail.com (J.N.); zohra.benzarti@dem.uc.pt (Z.B.); 2 Laboratory of Multifunctional Materials and Applications (LaMMA), Department of Physics, Faculty of Sciences of Sfax, University of Sfax, Soukra Road km 3.5, B.P. 1171, Sfax 3000, Tunisia

**Keywords:** CuO nanoparticles, green synthesis, *Opuntia ficus*-*indica*

## Abstract

This study investigates the structural, chemical, and morphological properties of CuO nanoparticles synthesized via a green synthesis route using *Opuntia ficus*-*indica* cladode extract, with a focus on the effects of stepwise versus direct calcination. Raman spectroscopy revealed the presence of CuO, Na_2_CO_3_, and Na_2_SO_3_, with the latter two being associated with elements inherited from the cactus extracts. XRD patterns confirmed the presence of crystalline CuO and Na_2_CO_3_ phases, with the low content of Na_2_SO_3_ inferred to be amorphous. Rietveld refinement estimated a CuO content of approximately 77% in the stepwise-calcined sample and 75% in the directly calcined sample, with lattice parameters closely aligning with reference values. SEM micrographs revealed a tendency for CuO nanoparticles to aggregate, likely due to high surface energy and interaction with the viscous plant extract used in the green synthesis. Crystallite size estimates, along with morphological observations, suggest that stepwise calcination enhances crystallinity and particle definition without altering the fundamental nanoparticle morphology. These findings highlight the influence of calcination method and natural extracts on the composition and morphology of green-synthesized CuO nanoparticles, offering insights into potential applications, namely in microelectronics, due to their promising dielectric properties.

## 1. Introduction

The synthesis and analysis of nanoparticles have gained considerable interest due to their fundamental and technological advantages [1]. Nanoparticles are commonly characterized as individual particles with a diameter between about 1 and 100 nm, exhibiting properties that are not found in bulk samples of the same material [2,3]. Metal nanoparticles are composed purely of metal precursors and can be monometallic, bimetallic, or polymetallic, while metal oxide nanoparticles are formed by combining a metal precursor with oxygen [4,5]. Metal and metal oxide nanoparticles display a wide variety of physicochemical properties, outperforming bulk materials in terms of chemical, electrical, optical, thermal, mechanical, electromagnetic, and surface characteristics. They additionally offer extensive surface areas, controlled size and shape, and easy surface modification. Alongside these advantages, they find applications in fields such as biomedicine, catalysis, environmental remediation, energy harvesting, molecular sensing, and more [5,6]. Metal oxide nanoparticles are a unique group separate from metal nanoparticles, and they are considered to be one of the most promising nanomaterials in various fields [7].

Currently, various metal oxide nanoparticles are being explored across numerous scientific fields, with copper oxide nanoparticles standing out due to their significant potential in both scientific and industrial applications. Copper (II) oxide (CuO) nanoparticles have been extensively studied because of their outstanding photocatalytic properties and resemblance to other metal nanoparticles [8].

Copper (II) oxide is a p-type semiconductor with a band gap ranging from 1.2 to 2.1 eV [9,10]. CuO has a number of unique characteristics including antimicrobial activity, efficient light absorption, and low cost, which position it as a promising metal oxide material [9]. CuO nanoparticles find applications in microwave irradiation, gas sensing, redox catalysis, microelectronics, energy storage, and various oxidation processes. They are also used in photoconductive and photothermal applications, showing effectiveness in adsorption processes [10,11,12,13].

The appeal of CuO nanoparticles is increased by their small size, high surface area, abundant raw materials, cost-effective production, and non-toxic nature [14].

Progress has been made in recent years to develop new synthetic methods for nano-sized materials, allowing precise control over their size, shape, and properties [7]. These methods fall into two main categories: (i) the bottom-up approach and (ii) the top-down approach [5].

The top-down method is a destructive process that involves breaking bulk materials into smaller units using a physical or chemical approach (such as lithography, ball milling, vapor and gas phase, evaporation–condensation, electro-deposition, pulsed laser ablation, arc discharge, sonication, and spray pyrolysis), which are then transformed into nanoparticles [6,15,16,17]. Although this method is simple, it usually requires expensive equipment and can lead to imperfections on the surface that may impact the physical characteristics and surface chemistry of the nanoparticles due to their elongated shape [5,6].

On the other hand, the bottom-up method is a constructive technique where atoms or molecules come together on their own to create bigger groups, subsequently shaping nanoparticles. This method involves techniques such as chemical vapor deposition, sol–gel, spinning, pyrolysis, and biological synthesis [3,6,15,16].

The sol–gel method, a bottom-up wet-chemical process, is commonly used in the production of different nanostructures, particularly metal oxide nanoparticles, in both traditional and commercial applications. It provides superb manipulation of particle size, high uniformity, and purity at cold temperatures [18,19]. The term refers to two fundamental concepts: sol and gel. A sol is a type of colloidal solution consisting of tiny particles dispersed in a liquid medium. A gel is a solid network with pores in three dimensions that encloses a liquid phase without interruptions. The sol–gel process consists of progressing from the sol phase to a gel-like continuous network, known as the gelling or viscous state, before reaching the final stage [20,21]. The sol–gel method provides numerous benefits, including simplicity, low cost, high homogeneity, and the production of nanoparticles with high purity. Furthermore, one of its key benefits is the lower processing temperature compared to conventional methods. Due to its exceptional properties, this method is capable of producing high-quality nanoparticles of uniform size on an industrial scale [6,19].

However, it is important to develop rapid, safe, non-toxic, and environmentally friendly methods for producing metal oxide nanoparticles [22]. Biosynthesis of CuO nanoparticles using eco-friendly techniques has gained increasing attention due to its numerous advantages over traditional chemical and physical methods, which often involve hazardous, expensive chemicals and/or complex equipment [23].

Green synthesis of nanoparticles through biological entities, such as plants [24], algae [25], bacteria [26], yeasts [27], fungi [28], and viruses [29], offers several benefits. While employing microorganisms to synthesize nanoparticles has advantages for the environment, there are drawbacks as well, including some bacterial strains’ toxicity and difficulties with the isolation and incubation procedures. On the other hand, the best technique for creating metal and metal oxide nanoparticles is still plant-based synthesis [30]. The use of plant extracts for the biosynthesis of nanoparticles offers numerous advantages, such as accessibility, biocompatibility, environmental friendliness, cost-effectiveness, high stability, and the absence of toxic chemicals. Plants can serve as both reducing and stabilizing agents, using water as a solvent and producing stable, cost-effective nanoproducts in large quantities [23,30,31]. Additionally, plant-based techniques rely on organic compounds like terpenoids, flavonoids, alkaloids, and phenolic compounds to control nanoparticle size, morphology, and performance through key factors like metal salt concentration, temperature, pH, and phytochemical composition [23,32,33].

Numerous studies into the synthesis of CuO nanoparticles from plant extracts have shown the effectiveness and sustainability of this method. One such instance, the ease of use and affordability of CuO nanoparticle synthesis using leaf extracts from *Phoenix dactylifera* L., has already been demonstrated [34]. In a different study, Bhatia et al. [35] used extracts from *Duchsnea indica* as stabilizing and reducing agents throughout the CuO nanoparticles synthesis process, achieving extremely stable particles with antibacterial activity. Additional investigations proved that plants such as *Daphnia magna* and *Ixora coccinea* could be used successfully, confirming the practicality of plant-based techniques [36,37].

Among potential plant sources, *Opuntia ficus*-*indica* offers a unique research opportunity in green nanoparticle synthesis. To the best of the author’s knowledge, it has yet to be explored for the green sol–gel route in synthesizing CuO nanoparticles.

*Opuntia ficus*-*indica* belongs to the *Cactaceae* family. Even though it originates from Mexico, it grows wild in arid and semi-arid regions of South and Central America, Africa, and the Mediterranean [38]. This prickly pear cactus has attracted global attention because of its ability to thrive where few other crops can, such as in Ethiopia, where successful crop growth is extremely challenging [39]. Its primary purpose of cultivation is for its fruit [40]; however, it also has healthcare uses due to its high content of polyphenols and its antioxidant, anti-inflammatory, and anxiolytic properties [39].

The cladodes of *Opuntia ficus*-*indica* exhibit antioxidant activity due to bioactive compounds, including polyphenolics (such as phenolics and flavonoids), vitamins (e.g., thiamine, riboflavin, niacin, and ascorbic acid), alkaloids, and carotenoids [41,42].

Additionally, a significant number of inorganic minerals are present in *Opuntia ficus*-*indica* cladodes. Among the macroelements, magnesium, sodium, potassium, and calcium are highlighted, while the trace elements include phosphorus, manganese, iron, zinc, and copper [43].

In this work, CuO nanoparticles were synthesized through a green sol–gel route using extracts from *Opuntia ficus*-*indica* cladodes, employing both a stepwise and a direct calcination process. Stepwise calcination enables better control over crystallite size and phase purity while promoting controlled grain growth [44]. Additionally, in green synthesis, organic compounds from plant extracts or other precursors often need to be gradually decomposed to avoid carbon residues or uncontrolled sintering. Lower initial temperatures can help remove volatile and organic components gently, preventing sudden decomposition.

The structure and morphology of the obtained powders were analyzed by Raman spectroscopy, X-ray diffraction, energy-dispersive spectroscopy, and scanning electron microscopy, while the dielectric characterization was performed through impedance spectroscopy analysis.

## 2. Materials and Methods

### 2.1. Materials

*Opuntia ficus*-*indica* cladodes were collected from an adult plant in a garden in Coimbra, Portugal, in the month of October.

For the synthesis of CuO nanoparticles, in addition to the *Opuntia ficus*-*indica* cladode extract, copper sulfate pentahydrate (CuSO_4_·5H_2_O, ≥99%, Labsolve, Warrington, UK) and sodium hydroxide (NaOH, 98%, Panreac, Barcelona, Spain) were used.

The deionized water used was self-produced using ultra-pure water equipment.

### 2.2. Preparation of the Opuntia ficus-indica Cladode Extract

*Opuntia ficus*-*indica* cladodes were thoroughly washed, first with tap water and then with deionized water, and, after the removal of the outer layer, were cut into small pieces. A mixture of deionized water and cladode pieces, in a 1:1 weight ratio, was blended for 30 s. The resulting slurry was then heated to 80 °C for 45 min under mechanical agitation. Afterward, the mixture was filtered through a 45 μm sieve and stored for future use.

### 2.3. Synthesis of the CuO Nanoparticles

The precursor solution was prepared by dissolving 5.00 g of CuSO_4_·5H_2_O in 100 mL of deionized water. Subsequently, 125 mL of plant extract was added to this solution dropwise. The mixture was stirred for 2 h at 70 °C. While maintaining heating and stirring conditions, an aqueous NaOH solution (0.08 wt%) was added, also dropwise, raising the pH from 2.62 to 12.70. The mixture was kept under these conditions for 45 min, during which the color changed from green to brick orange. After sedimentation overnight, the material was dried at 150 °C for 4 h.

For the calcination, two different approaches were followed: four cycles of heat treatment, each for 2 h followed by cooling, with a heating rate of 15 °C/min at 300, 500, 600, and 700 °C, respectively, and a single heat treatment at 700 °C for 2 h, also with a heating rate of 15 °C/min.

**Figure 1:**
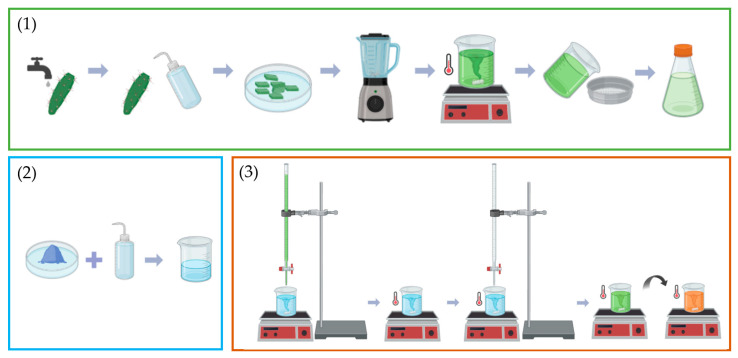
Schematic representation of the preparation of *Opuntia ficus*-*indica* cladode extract, step (1), and the synthesis of CuO nanoparticles, steps (2) and (3). (Created in BioRender. Devesa, S. (2024) https://BioRender.com/b68l138).

Figure 1 shows the flowchart of the steps involved in the synthesis of CuO nanoparticles.

### 2.4. Characterization Techniques

X-ray diffraction (XRD) analyses were carried out using a Philips X’Pert PRO diffractometer (Almelo, The Netherlands) with Cu Kα (λ = 1.54060 Å) radiation, operating at 35 kV and 30 mA. The tests utilized a theta/2 theta geometry, with a step size of 0.02°.

Raman spectroscopy measurements were executed with a Renishaw inVia™ (Wotton-under-Edge, UK) confocal Raman spectrometer, employing a 532 nm green laser.

The morphology of the sintered powders and the internal tissue of *Opuntia ficus*-*indica* cladodes was examined by scanning electron microscopy (SEM), using a Hitachi SU3800 microscope (Tokyo, Japan) and a Zeiss Merlin microscope (Zeiss, Oberkochen, Germany), respectively, both in secondary electron mode. The chemical composition of the internal tissue of *Opuntia ficus*-*indica* cladodes was analyzed via energy-dispersive X-ray spectroscopy (EDS) using a Bruker Nano system (Berlin, Germany), set at an accelerating voltage of 10 keV.

The electrical measurements in the frequency range from 100 Hz to 100 MHz were performed using the precision impedance analyzer Agilent 4294A (Santa Clara, CA, USA) in the *C_p_* − *R_p_* configuration at room temperature.

## 3. Results

### 3.1. Opuntia ficus-indica

**Figure 2:**
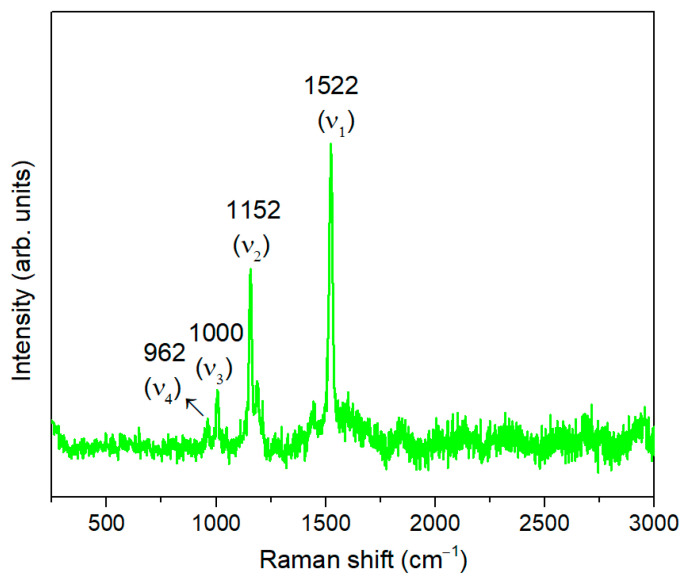
Raman spectrum of the internal tissue of the cladodes of *Opuntia ficus*-*indica*.

The Raman spectrum of the internal tissue of the *Opuntia ficus*-*indica* cladodes is shown in Figure 2.

The Raman spectrum is dominated by three bands, centered at 1522, 1152 and 1000 cm^−1^, that are well aligned with the three prominent bands assigned to carotenoids [45]. The band centered at 1522 cm^−1^, *v*_1_, is due to the stretching vibrations of the conjugated C=C backbone of carotenoids. The *v*_2_ band, centered at 1152 cm^−1^, has been widely assigned to a combination of C–H in-plane bending and C–C stretching vibrations of the polyene chain. The third most prominent band, *v*_3_, centered at 1000 cm^−1^, arises from C–CH_3_ in plane-rocking deformations of methyl side chains coupled to C–C bonds [45,46].

Below 1000 cm^−1^, there is an additional band, centered at 962 cm^−1^, *v*_4_, which arises from out-of-plane motions of the H nuclei on the conjugated chain. Although relatively weak, it can increase in intensity if there are distortions in the plane modes of the carotenoids, for example, due to steric hindrance within a protein binding pocket [45]. A few other weaker features, resonantly enhanced in the 532 nm spectra, also contribute to the Raman spectrum and can be found in the region > 2000 cm^−1^. These weaker features are overtone and combination bands which arise from the total symmetric character of the carotenoid vibrations (*v*_1_, *v*_2_ and *v*_3_) [45,47].

**Figure 3:**
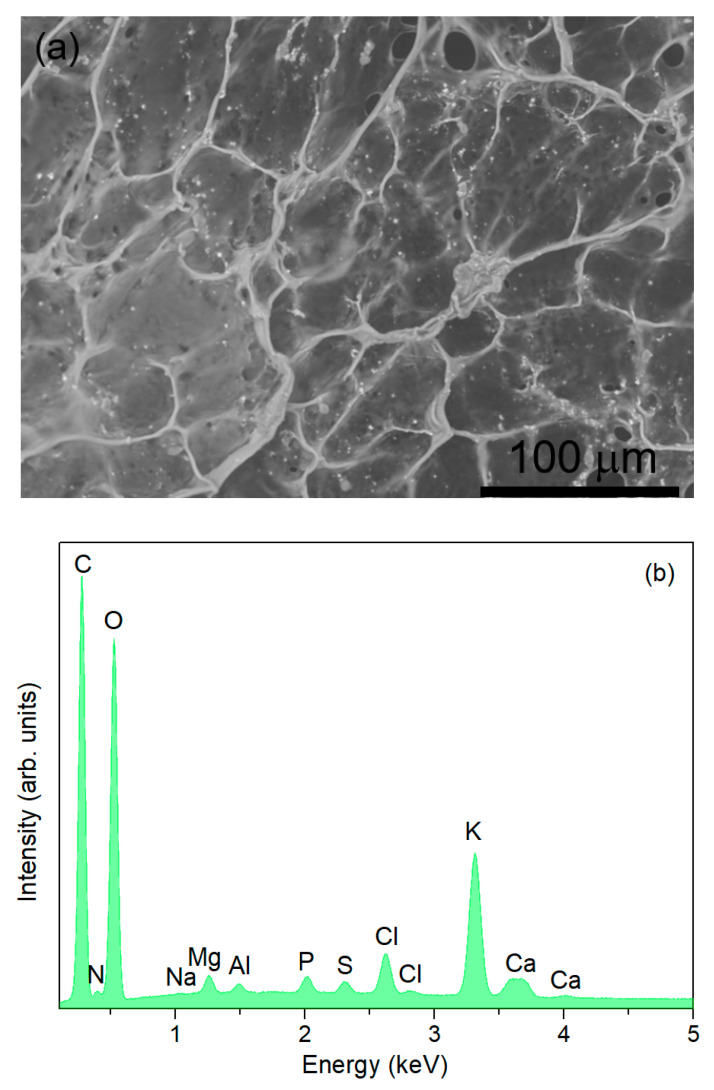
(**a**) SEM micrograph taken at a magnification of 1000×; (**b**) EDS spectrum of the internal tissue of the *Opuntia ficus*-*indica* cladodes.

Figure 3a shows the SEM micrograph of the internal tissue of *Opuntia ficus*-*indica* cladodes, revealing elongated and interconnected fibers that form structures with smooth surfaces, characteristic of cellulose. Surrounded by these structures, a heterogeneous, fibrous morphology is observed, indicative of lignocellulosic material. These observations align with the known presence of cellulose, hemicellulose, and lignin as structural carbohydrates in *Opuntia ficus*-*indica* cladodes [48,49,50].

**Figure 4:**
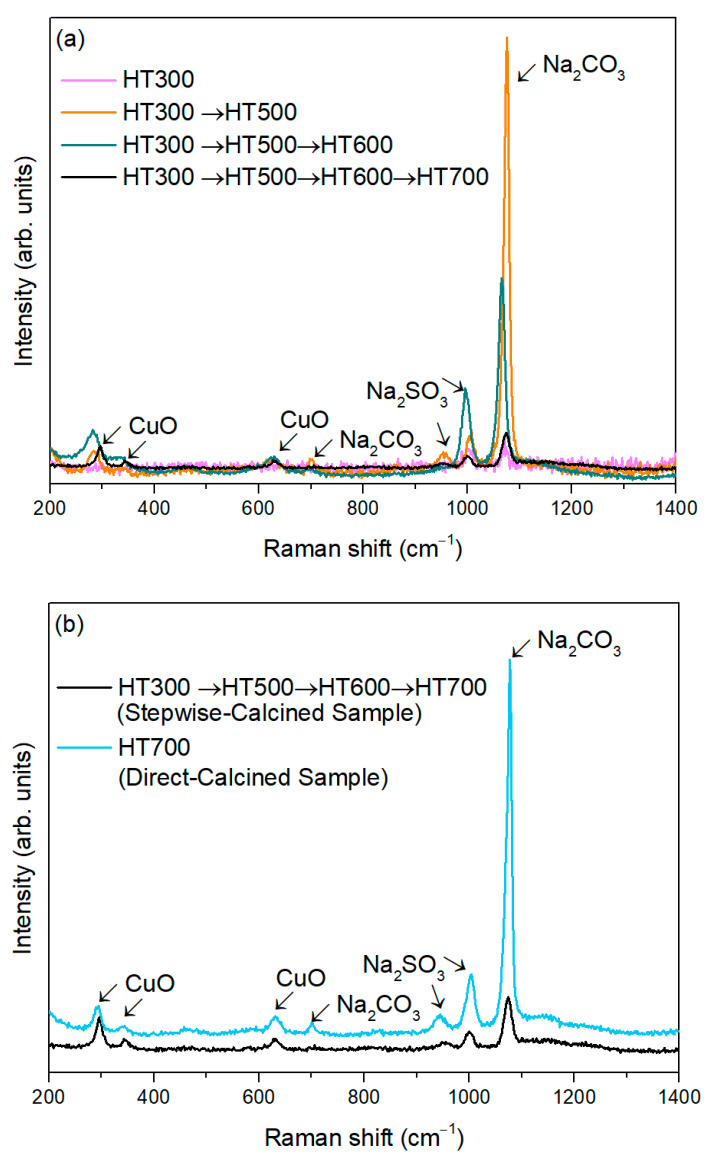
Raman spectra of (**a**) the stepwise-calcined sample and (**b**) the directly calcined sample, with the bands assigned to CuO, Na_2_CO_3_ and Na_2_SO_3_.

The corresponding EDS spectrum of the internal tissue of *Opuntia ficus*-*indica* cladodes is depicted in Figure 4b, showing the presence of carbon, oxygen, sodium, magnesium, aluminum, phosphorus, sulfur, chlorine, potassium, and calcium.

These findings exhibit some variation compared to previous reports, including those by [43,49], which themselves do not coincide, as cladode chemical composition varies with factors like soil characteristics (pH, salinity, structure), climate conditions during the growing season, and plant age [43,50]. Additionally, the identification of trace elements can be influenced by the resolution of the EDS equipment.

### 3.2. CuO Nanoparticles

Figure 4a displays the Raman spectra of the synthesized powders after each heat treatment cycle. After the first annealing, performed at 300 °C, CuO bands are absent, but a band at 1073 cm^−1^, attributed to Na_2_CO_3_, is observed. This is the most intense Raman band for Na_2_CO_3_ and corresponds to the ν_1_ symmetric stretching vibration of the carbonate group [51]. The second cycle, made at 500 °C, significantly increases the intensity of this band and reveals a new Na_2_CO_3_ band at 702 cm^−1^, corresponding to the *v*_4_ asymmetric bending mode [51]. Additionally, two of the three bands attributed to CuO begin to emerge, indicating the onset of CuO crystallization. After the two remaining cycles, at 600 and 700 °C, all three CuO bands are clearly visible.

CuO has a monoclinic structure with a $C_{2h}^{6}$ space group with two molecules per primitive cell, yielding zone center Raman active modes of Γ_RA_ = 4A_u_ + 5B_u_ + A_g_ + 2B_g_. These include three acoustic (A_u_ + 2B_u_), six infrared-active (3A_u_ + 3B_g_), and three Raman-active modes (A_g_ + 2B_g_). The known Raman bands of CuO are Ag (295 cm^−1^), B1g (345 cm^−1^), and B2g (629 cm^−1^), which are in good agreement with the literature [52]. In the A_g_ and B_g_ modes, only oxygen atoms move: A_g_ mode displacements are along the b-axis, while B_g_ modes are perpendicular to it [53].

After the fourth heat treatment cycle, the CuO bands are better defined, suggesting improved crystallinity and reduced structural disorder [54]. Also, the Ag band becomes sharper and shows a slight blue shift with the heat treatment cycles. This can be related to the grain size increase that is expected from the increase in the heat treatment temperature. Similar results were reported by Xu et al. [55]. Additionally, the intensity of the most prominent Na_2_CO_3_ band decreases, and it undergoes a slight shift, while the 702 cm^−1^ band disappears entirely, likely indicating the structural decomposition of Na_2_CO_3_.

The bands centered at 948 and 1004 cm^−1^ can be attributed to Na_2_SO_3_, corresponding to the anti-symmetric and symmetric SO_3_ stretching vibrations, respectively [56]. The intensity evolution of these peaks is similar to that observed for Na_2_CO_3_, indicating that the final heat treatment cycle also promotes its decomposition. Both phases are associated with elements inherited from the cactus extracts.

The sample obtained after the four heat treatment cycles is hereafter identified as the stepwise-calcined sample.

Figure 4b shows this stepwise-calcined sample as well as the directly calcined sample, which corresponds to a single heat treatment performed at 700 °C for 2 h. The CuO bands are better defined, and the decreased intensity or disappearance of foreign bands suggests the enhanced potential of the stepwise-calcined sample.

**Figure 5:**
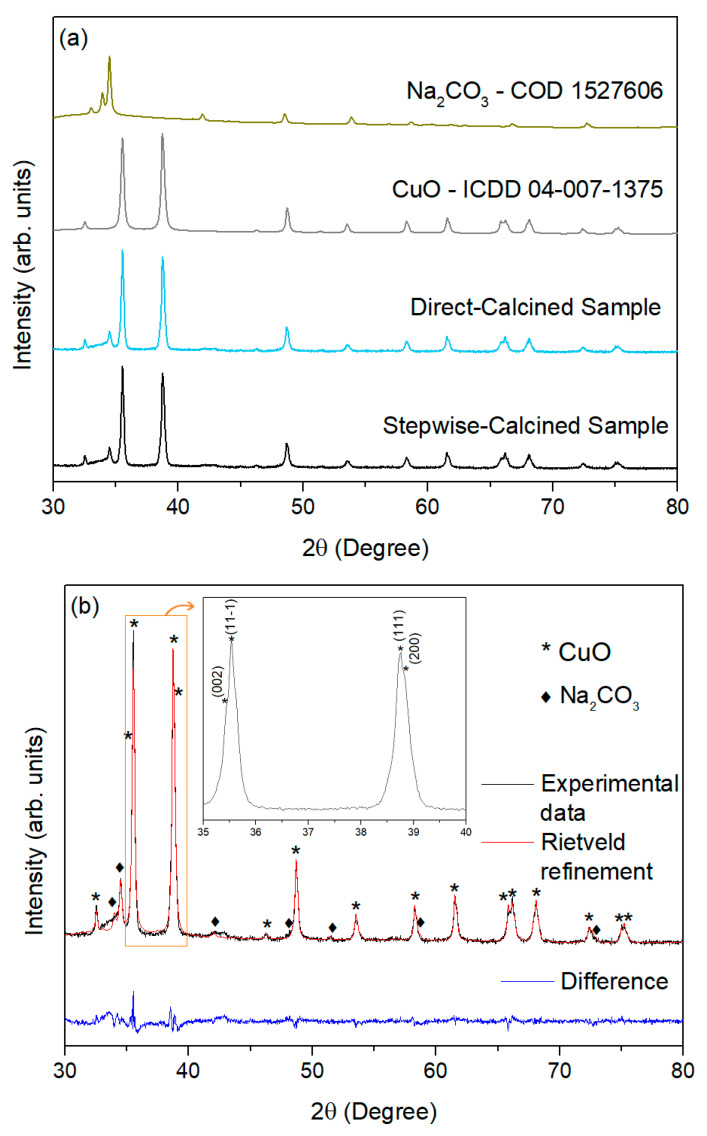
(**a**) XRD diffractogram of the stepwise-calcined and directly calcined samples. (**b**) Measured and calculated XRD diffractograms of the stepwise-calcined sample (inset: magnification of the two most intense peaks of CuO with their corresponding Miller indices).

Figure 5a shows the XRD patterns of the stepwise-calcined and directly calcined samples, along with references for CuO [57] and Na_2_CO_3_ [58]. All observed peaks can be attributed to these two crystalline phases, suggesting that the Na_2_SO_3_ phase, whose presence was confirmed by the Raman analyses, exists in an amorphous or quasi-amorphous state with a negligible amount in the sample. This conclusion is supported by the absence of a characteristic hump for amorphous material in the XRD diffractograms. Regarding CuO, the narrow peaks indicate good crystallinity, with no noticeable differences between the two samples.

To quantify the chemical composition of the crystalline phases present in these two samples, Rietveld refinement was applied using the software Profex (Version 5.0.1) [59]. Figure 5b displays the measured and calculated diffractograms and the diffraction patterns of the stepwise-calcined sample, along with the peak assignments for each phase. The inset highlights the more intense peaks with their corresponding Miller indices.

Table 1 shows the percentage of each crystalline phase, confirming the results of the Raman analysis. The estimated cell parameters of the CuO phase are also presented and align well with the aforementioned reference [57], demonstrating the consistency of the results. Finally, the crystallite size was determined using the Rietveld method, yielding a value of 52.9 nm for the stepwise-calcined sample and 43.0 for the directly calcined sample.

The Rietveld fitting parameters, also included in Table 1, indicate the good quality of the fitting [60].

**Figure 6:**
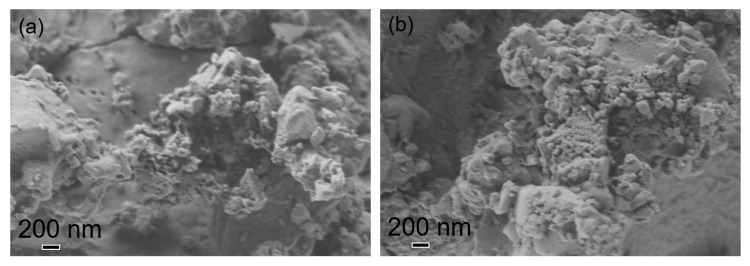
SEM micrographs of the (**a**) stepwise-calcined and (**b**) directly calcined samples, taken at a magnification of 30 k×.

The morphological properties of the prepared samples were observed using SEM, with the corresponding micrographs presented in Figure 6. These images show that only a few nanoparticles with a spherical shape were synthesized. Some nanoparticles are well separated, while most are present in an agglomerated form.

This agglomeration is typically attributed to the large surface area and high surface energy of nanoparticles [61,62], which are further enhanced when the synthesis is carried out in an aqueous medium [63]. Additionally, green-synthesized nanoparticles tend to have a higher surface area and adhere strongly to each other due to the viscous nature of plant extracts [62], leading to a tendency to form asymmetrical clusters [64].

The formation of larger, bulky particles in the synthesized CuO may be attributed to the reduction in the grain boundary area as the growing grains disturb each other and decrease the crystal surface energy during aggregation under calcination. This process reduces the pore volume, leading to compact shrinkage as the calcination temperature increases [62,65]. No observable differences were found between the stepwise and direct calcination processes applied.

### 3.3. Dielectric Properties

The dielectric permittivity is represented by [66]

(1) $$\varepsilon^{*}=\varepsilon^{\prime }+j\varepsilon^{\prime \prime }$$

The first term, the real part or dielectric constant, describes the stored energy, while the second term, the imaginary part or dielectric loss, represents the dissipated energy [67].

The real and imaginary parts of permittivity were calculated using the following relations [68]:

(2) $$\varepsilon^{\prime }=\frac{d}{A}\frac{C_{p}}{\varepsilon_{0}}$$

(3) $$\varepsilon^{\prime \prime }=\frac{d}{A}\frac{1}{\omega {R_{p}\varepsilon }_{0}}$$

where *C_p_* and *R_p_* are the measured capacitance and resistance, respectively, *ω* is the angular frequency, *d* is the sample thickness, *A* is the electrode area, and *ε*_0_ is the vacuum permittivity (8.8542 × 10^−12^ F/m).

**Figure 7:**
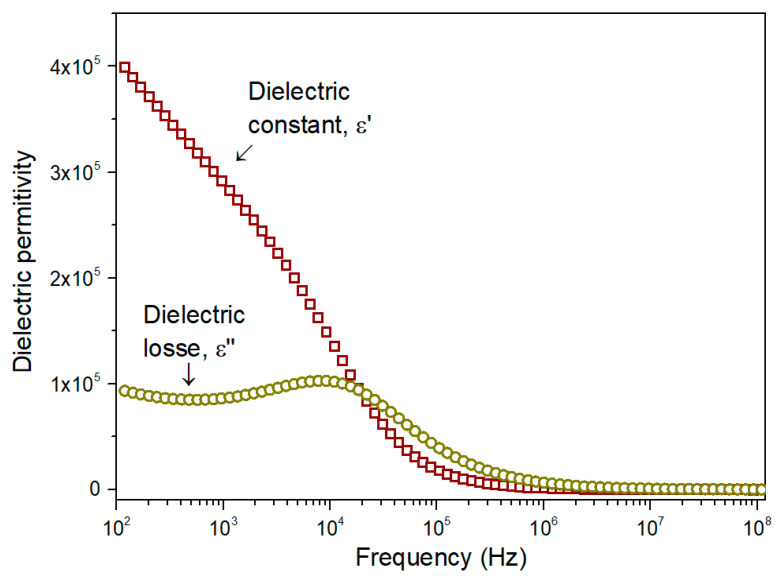
Dielectric constant and dielectric loss, as a function of frequency, obtained at room temperature, for the stepwise-calcined sample.

Figure 7 shows the variation in the dielectric constant and dielectric loss as a function of frequency, at room temperature, for the stepwise-calcined sample, which presents a higher percentage of CuO and enhanced crystallinity.

The dielectric constant initially decreases significantly at lower frequencies, but this decline becomes more gradual at higher frequencies, eventually stabilizing and becoming nearly frequency-independent. This behavior is typical of dielectric materials and arises because, as the frequency of the applied electric field increases, the polarization mechanisms cannot keep up with the rapid changes. Consequently, their contribution to the overall dielectric constant diminishes [69].

The dielectric dispersion behavior can be explained using the Maxwell–Wagner model for a homogeneous double-layer structure, in line with Koop’s phenomenological theory. This model suggests that the dielectric material consists of a conducting layer formed by the grains, which is surrounded by a less conductive layer corresponding to the grain boundaries, with these different layers contributing to the overall dielectric response [67].

The frequency dependence of the dielectric loss, as shown in Figure 7, exhibits a peak, indicating the presence of a relaxation phenomenon. Additionally, up to 20 kHz, the dielectric loss is significantly lower than the dielectric constant, implying that the energy dissipated is less than the energy stored. This suggests that the material has good potential for energy storage applications.

Comparing the dielectric properties of the studied sample with the results reported by Chand et al. [13], who used different copper precursors in the conventional sol–gel method, it is clear that, particularly in the abovementioned frequency range, the sample presents a significantly higher dielectric constant and enhanced potential for energy storage.

Regarding the results reported by Vindhya et al. [70], who used *Annona muricata* leaf extracts in a green synthesis route, it is evident that, especially within the frequency range discussed, the dielectric constant of the studied sample is also significantly higher.

Based on these results, it is valid to infer that, as previously reported, the inherited phases from the plant extracts used in green synthesis routes can positively impact the performance of the obtained material.

## 4. Conclusions

CuO nanoparticles were successfully synthesized via a green synthesis route using *Opuntia ficus*-*indica* cladode extract, demonstrating that this extract can act as a natural reducing agent, replacing traditional chemical reagents to reduce metal ions to lower valence states. Raman spectroscopy revealed the presence of CuO, Na_2_CO_3_, and Na_2_SO_3_, with the latter two being associated with elements inherited from the cactus extracts. XRD analysis confirmed the crystalline phases of CuO and Na_2_CO_3_, while Na_2_SO_3_ appeared to be present in a low concentration, likely in an amorphous form. Rietveld refinement indicated that the CuO content was slightly higher in the sample subjected to stepwise calcination compared to the directly calcined sample.

Materials synthesized via green routes often retain structures and functional groups inherited from their organic precursors. These structures can impart unique mechanical, chemical, electrical, and optical properties to nanomaterials, making them useful in diverse fields, from biomedical applications to electronics.

The morphological characterization revealed a few spherical nanoparticles and agglomeration, as well as bulky particles, which could be attributed to several factors, some inherent to the biosynthetic process. The viscous nature of the plant extract and the presence of hydroxyl groups likely promote particle cohesion, while van der Waals forces contribute to further agglomeration, binding the particles closely together. The formation of bulky particles is a consequence of the calcination process, with both stepwise and direct calcination approaches showing similar outcomes.

The dielectric characterization of the stepwise-calcined sample showed the typical behavior of dielectric materials, with potential for energy storage applications.

## Figures and Tables

**Table 1:** Chemical composition, Rietveld fitting parameters, and lattice parameters and crystallite size estimated for the CuO phase.

Sample	Stepwise-Calcined Sample		Directly Calcined Sample		
Crystalline phases	CuO	Na_2_CO_3_	CuO	Na_2_CO_3_	
Content (wt%)	77.15	22.85	75.10	24.92	
Rietveld fitting parameters	R_wp_ = 3.90 R_exp_ = 2.46	GoF = 1.59	R_wp_ = 3.65 R_exp_ = 1.76	GoF = 2.07	
Lattice parameters	a (nm)	b (nm)	c (nm)	β (°)	Crystallite size (nm)
CuO stepwise-calcined sample	0.46925	0.34228	0.51342	99.54	(111) 52.9 ± 0.7
CuO Directly calcined sample	0.46896	0.34226	0.51322	99.51	(111) 43.0 ± 0.5
CuO Reference 04-007-1375 [57]	0.46837	0.34226	0.51288	99.54	---------

## Data Availability

The original contributions presented in this study are included in the article. Further inquiries can be directed to the corresponding author.
